# Respiratory health screening for opiate misusers in a specialist community clinic: a mixed-methods pilot study, with integrated staff and service user feedback

**DOI:** 10.1136/bmjopen-2016-012823

**Published:** 2016-10-14

**Authors:** Caroline Anne Mitchell, Alice Pitt, Joe Hulin, Rod Lawson, Fleur Ashby, Ivan Appelqvist, Brigitte Delaney

**Affiliations:** 1 Academic Unit of Primary Care, The University of Sheffield, Sheffield, UK; 2 School of Health and Related Research, The University of Sheffield, Sheffield, UK; 3 Sheffield Teaching Hospitals NHS Foundation Trust, Sheffield, UK; 4 Barnsley NHS Trust/Phoenix Futures, Wakefield, UK

**Keywords:** Opiate Addiction, Substance Abuse, Chronic Obstructive Pulmonary Disease

## Abstract

**Objectives:**

Increased rates of illicit drug inhalation are thought to expose opiate misusers (OMUs) to an enhanced risk of respiratory health problems. This pilot study aimed to determine the feasibility of undertaking respiratory screening of OMUs in a community clinic.

**Setting:**

Single-centre UK community substance misuse clinic.

**Participants:**

All clinic attendees receiving treatment for opiate misuse were eligible to participate. 36 participants (mean age=37) were recruited over a 5-week period. The sample included 26 males and 10 females.

**Outcome measures:**

Spirometry without bronchodilation; health related quality of life EQ-5D-3L; Asthma Control Test; Mini Asthma Quality of Life; Clinical COPD Questionnaire and the Treatment Outcome Profile were used to assess the respiratory health of participants. Findings were discussed with staff and service users in 2 patient and public involvement events and feedback was analysed thematically.

**Results:**

34 participants reported that they had smoked heroin. 8 participants diagnosed with asthma, scored under 13 on the Asthma Control Test, suggesting poorly controlled asthma. Participants (n=28), without a diagnosis of asthma completed the Lung Function Questionnaire. Of these, 79% produced scores under 18, indicating symptoms associated with the development of chronic obstructive pulmonary disease. Spirometry showed 14% of all participants had forced expiratory volume in 1 s/forced vital capacity <0.7 (without bronchodilator), indicating potential obstructive lung disease. Feedback from service users and staff suggested a willingness and capacity to deliver respiratory health screening programmes. Insight towards the difficulties service users have in accessing services and the burden of respiratory health was also provided.

**Conclusions:**

It is feasible to undertake respiratory health screening of OMUs in a community clinic. Larger screening studies are warranted to determine the prevalence of respiratory health problems in this population. Research regarding asthma medicines adherence and access to healthcare among OMUs is also required.

**Strengths and limitations of this study**

- Owing to the small sample size, the quantitative findings from this pilot study, in relation to the prevalence of poor respiratory health in this population, have to be approached with caution.
- Integrated patient and public involvement feedback allowed exploration of the burden of respiratory disease in this population, the barriers to respiratory care and potential solutions for how to overcome these barriers from a service user and practitioner perspective.
- This study investigated the contribution of asthma control to poor respiratory health in opiate misusers through the use of validated self-report measures.
- There is an ongoing debate over the appropriateness of the use of the Lung Function Questionnaire (LFQ) and spirometry testing as population screening tools for chronic obstructive pulmonary disease.

## Background

Owing to the potential consequences of injecting behaviours, such as bloodborne virus transmission (chronic liver disease, HIV/AIDS) and venous ulceration post-venous thromboembolism (VTE), there was a gradual increase in the inhalation of opiates from the early 1990s. Although there is a paucity of evidence regarding rates of opiate inhalation in recent years, it has been reported that by the late 1990s, this was the most frequent form of opiate use.^1^ However, there are now growing concerns over the impact the inhalation of opiates may have on an individual's respiratory health. For example, there is evidence that heroin inhalation can trigger asthma exacerbations^2–4^ and findings from the UK Review of Asthma Deaths (2014) show that substance misuse was implicated in 6% of deaths.^5^

Inhalation of opiates has been linked to early-onset chronic obstructive pulmonary disease (COPD),^6^ which is the fifth biggest killer in the UK.^7^ Furthermore, two-thirds of those affected by COPD are thought to be undiagnosed.^8^ The independent effects of heroin inhalation on the development of COPD remain difficult to determine from previous research, due to a failure to control for tobacco smoking, which is also highly prevalent in this population.^6^ However, opiate misusers (OMUs) remain a high-risk group, and the contribution of circulatory and respiratory disease to premature mortality of among opioid misusers in England has been recently highlighted.^9^ In this study, data from a cohort of 198 247 OMUs suggested that respiratory system diseases accounted for 7% of all deaths in this population. A recent screening study across three UK Crime Reduction Initiative (CRI) services, also demonstrated a high prevalence of COPD in relation to opiate inhalation, with findings from spirometry testing suggesting that a minimum of 28% of current or former heroin smokers demonstrated evidence of airflow obstruction consistent with COPD. It was also reported that COPD was present in service users at a relatively young age. The authors concluded that the screening of respiratory health of OMUs in community services should be explored further.^10^

Research has suggested that OMUs often fail to engage with primary care services and display an over-reliance on emergency departments,^11^ contributing to a significant economic burden on healthcare systems.^12^ Factors such as early-onset drug/alcohol-related cognitive impairment, treatment concordance and poor social support compound the difficulty in optimising proactive medical care for OMUs.^13^ Further research exploring the potential benefit of screening programmes targeting high-risk groups is advocated through current guidelines proposing that the early diagnosis of COPD can significantly slow lung function decline and increase the duration a person can experience an active lifestyle.^14^

The aim of the current study was to determine the prevalence of diagnosed and undiagnosed respiratory health problems and feasibility of undertaking respiratory screening of OMUs in a community clinic, in order to define recruitment and outcome measure parameters for future research. We undertook spirometry and administered validated lung health screening, condition-specific and health-related quality of life questionnaires. The study integrated patient and practitioner group feedback into the research process, in order to identify the research priorities of importance to service users and practitioners and to collaboratively address research study practicalities and questions arising from the pilot study.

## Methods

### Pilot study

Opportunistic sampling methods were employed twice weekly to recruit participants attending a community substance misuse clinic, from 3 February 2015 to 14 March 2015. All service users were eligible for inclusion in the study if they had ever smoked or injected opiates. This broad inclusion criterion was used to gain an indication of the prevalence of smoking and injecting behaviour in this group. Study recruitment was advertised through the display of posters within waiting rooms at the community substance misuse clinic and the distribution of information leaflets to the clinic staff 2 weeks prior to the study start date. Participants were offered a £10 supermarket voucher to cover their time commitment and travel costs.

Current National Institute for Health and Care Excellence (NICE) guidelines,^15^ ^16^ a recent evaluation of screening of COPD against the National Screening Committee (NSC) criteria,^17^ and two monographs^18^ ^19^ on recommended outcome measures for asthma and COPD, respectively, were used to determine screening processes and condition-specific outcome measures for the study. The outcome measures were also reviewed by an expert panel, which included a consultant psychiatrist in substance misuse, a consultant respiratory physician, a professor in respiratory medicine and a general practitioner with a special interest in substance misuse, in order to ensure that the following measures were research and clinically appropriate.

1. Study-specific sociodemographic data questionnaire:
  Patient-completed questionnaire used to capture data on: gender, age, smoking status, current illicit and prescribed drug use.
  2. Health-related quality of life EQ-5D-3L questionnaire:^20^ A generic measure of health status.
  3. The LFQ:^21^ A patient-completed screening tool, to be used in primary care settings, to identify patients who are appropriate for spirometry testing for airflow obstruction.
  4. The Asthma control test (ACT):^22^ The ACT has a total score up to 25 and assesses the impact and control of a patient's asthma over the past 4 weeks. A score <20 indicates that asthma may not have been well controlled in the previous 4 weeks.
  5. Mini Asthma Quality of Life Questionnaire (Mini—AQLQ):^23^ A validated short version of the original Asthma Quality of Life Questionnaire (AQLQ),^24^ which measures the impact of asthma on an individual's quality of life and has an overall score, and four dimensional scores (‘symptoms’; ‘activity’; ‘emotional’; ‘environmental’). The highest Mini-AQL score of 7.00 indicates no impairment due to asthma; scores below 7 indicate some impairment; scores of 1.00 indicates severe impairment and 4.00 indicates a moderate degree of impairment of quality of life.
  6. Clinical COPD Questionnaire (CCQ):^25^ A validated questionnaire used to measure the symptoms and functional status of patients with COPD.
  7. Treatment Outcome Profile (TOP):^26^ The TOP is the standard UK outcome measure for substance misusers in treatment and is completed by clinical staff with OMUs at appointments. The data from this questionnaire were collected, with consent, on a separate visit by a member of the research team (AP), from the patient clinical record. This information was triangulated with the sociodemographic information collected in order to reduce participant questionnaire burden relating to additional psychosocial, drug use and physical health information.
  8. Spirometry: Spirometry is used to monitor the severity of lung conditions and is used as a screening tool for COPD. Postbronchodilator spirometry testing was not performed, as dispensing inhaled medication was beyond the scope of the feasibility study. NICE guidance was used to categorise airways obstruction as measured without bronchodilation.^15^ ^16^

The overall relevant questionnaire and spirometry results were discussed with the participant, and dependent on these results, they were advised to attend their GP for follow-up if there were any abnormalities. A copy of the individual's spirometry and questionnaire results, with an information sheet regarding the study, were posted to each patient's GP.

### Patient and public involvement

Following study completion, the results of the feasibility study were also discussed with staff at the community substance misuse clinic and service users in two separate patient and public involvement (PPI) events, to facilitate a discussion about future priorities and research practicalities. The service user forum included service users currently on treatment and undertaking a recovery rehabilitation programme. The forum for healthcare professionals included members of staff at the community clinic, including a consultant psychiatrist, mental health nurses and key workers.

## Results

The recruitment target of 30 participants was exceeded within 5 weeks: 36 participants agreed to take part in the study. The outcome measures administered to each participant were dependent on self-reported previous diagnosis of asthma or COPD.

**Figure 1:**
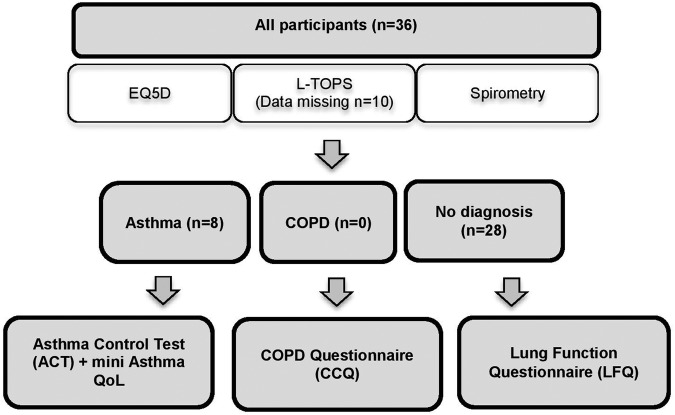
Participant recruitment and flow chart. COPD, chronic obstructive pulmonary disease; TOPs, Treatment Outcome Profiles.

Figure 1 summarises participant flow according to self-reported prior lung disease and completion of screening questionnaire for those with no prior lung disease diagnosis. All participants completed the health-related quality of life EQ-5D-3L questionnaire and underwent spirometry. TOP data were collected for 26 participants, with no data available from the remaining 10 participants. Of the original 36, 8 participants had a diagnosis of asthma and completed the asthma-specific questionnaires (ACT, Mini-AQL), nobody reported a diagnosis of COPD and the remaining 28 participants had no prior diagnosis of lung disease and thus completed the ‘case finding questionnaire, the LFQ’.

### Sample characteristics

In common with the reported predominance of male OMUs in treatment, there were a greater number of male (26/36) than female (10/36) participants. The mean age of participants was 37 years (range 24–53 years; table 1).

**Table 1:** Participant characteristics (n=36)

Age (years)	Mean (SD)	Range							
	37 (6.4 7)	24–53							
**Gender**	**Male**	**Female**							
	26	10							
**Work status**	**Employed n (%)**	**Unemployed n (%)**	**Registered disabled n (%)**	**Missing n (%)**					
	2 (5.6)	32 (88.9)	1 (2.8)	1 (2.8)					
**Living arrangements**	**Living alone n (%)**	**Living with parents n (%)**	**Living with partner n (%)**	**Hostel n (%)**	**Temporary n (%)**	**Living with children n (%)**	**Shared housing n (%)**	**NFA n (%)**	**Missing data n (%)**
	5 (13.9)	9 (25)	5 (13.9)	4 (11.1)	2 (5.6)	3 (8.3)	4 (11.1)	3 (8.3)	1 (2.8)

NFA, no fixed abode.

Table 2 summarises data collected regarding smoking status, drug use, influenza vaccination uptake and history of respiratory problems. Data provided on inhaled drug use relate to all history and not solely to participant's current inhaled drug use status. Almost all participants smoked heroin, crack cocaine, tobacco or cannabis (table 2).

**Table 2:** Self-reported smoking status, drug use, influenza vaccination uptake and respiratory problems (n=36)

Smoking status	Current smoker n (%)	Ex-smoker n (%)	Never smoked n (%)
	35 (97)	1 (3)	0 (0)
**Years smoked**	**Mean (SD)**	**Range**	
	24 (7.60)	2–40	
**Cigarettes smoked per day**	**Mean (SD)**	**Range**	
	15 (10.60)	0–50	
**Inhaled drug use**	**Heroin n (%)**	**Cocaine n (%)**	**Cannabis n (%)**
	34 (94)	33 (92)	31 (86)
**Influenza vaccination history (previous 2 years)**	**Received influenza vaccination n (%)**	**Failed to receive influenza vaccination n (%)**	
	9 (25)	27 (75)	

### Health-related quality of life EQ-5D-3L questionnaire

Findings from the generic health-related quality of life measure showed that the majority of participants reported to suffer from anxiety and or/depression, with 58% of the sample reporting having extreme problems in this instance (table 3). More than half of the participants also reported having problems with mobility and pain/discomfort.

**Table 3:** Self-reported health status (n=36)

	Mobility	Self-care	Usual activities	Pain/discomfort	Anxiety/depression
No problems n (%)	13 (36)	25 (69)	22 (61)	14 (39)	5 (14)
Some problems n (%)	23 (64)	11 (31)	14 (39)	17 (47)	10 (28)
Extreme problems n (%)	0 (0)	0 (0)	0 (0)	5 (14)	21 (58)

### Spirometry

All participants had screening spirometry with 5/36 (14%) having forced expiratory volume in 1 s/forced vital capacity <0.7 (without bronchodilator). Two of these five patients had asthma, but without bronchodilation, we cannot determine if there is now a degree of COPD within this group.

### The Treatment Outcome Profile

TOP data could be acquired for 26/36 participants included in the study. Some issues were identified with the consistency of the data, particularly in relation to the discrepancies between measurements of injecting status. In this instance, conflicting data were collected from service users in relation to the items measuring current injecting status, injecting status within the past 30 days and total number of days injecting in this period. This suggests the scale may not demonstrate sufficient internal reliability, and due to such issues, the TOP data have not been presented in full.

### Patients with no prior diagnosis of COPD or asthma

#### Lung Function Questionnaire

**Figure 2:**
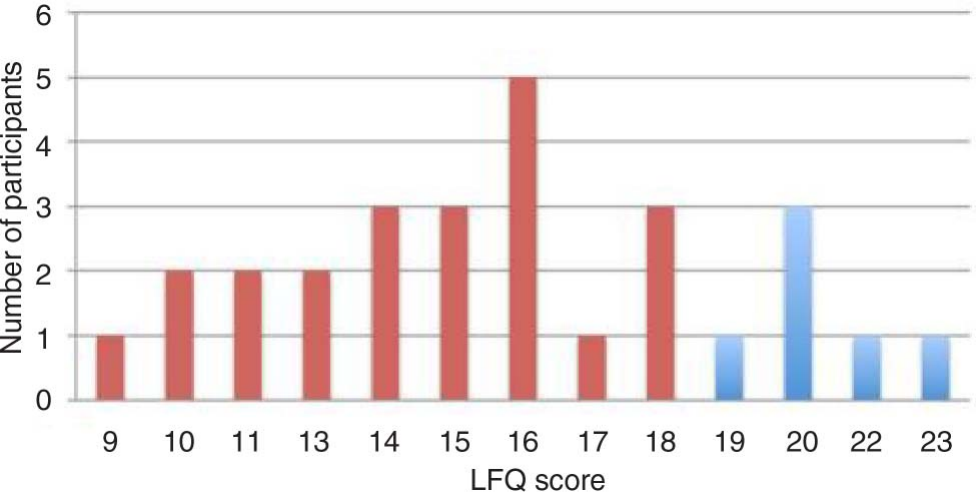
LFQ scores from participants with no prior diagnosis of COPD or asthma (n=28). COPD, chronic obstructive pulmonary disease; LFQ, Lung Function Questionnaire.

Twenty-eight participants (78%) reported no prior diagnosis of COPD or asthma and completed the LFQ. The mean LFQ score was 15.68 (SD=3.63) and 22 participants (79%) scored 18 or less, suggesting these participants had symptoms associated with a risk of developing COPD (figure 2).

### Participants with prior diagnosis of asthma

Eight of 36 participants self-reported a diagnosis of asthma. All asthmatics reported significant respiratory symptoms, poor control of their asthma and excessive daily use of salbutamol (β agonist) inhalers. None of the participants with asthma was prescribed an inhaled corticosteroid. The potential impact of comorbid asthma on service users was also highlighted in the following free-text response on a questionnaire:

…sometimes I am frightened to go to sleep at night (because of my breathing)…

#### Asthma control (ACT)

On average, patients reported a score of 8.86 (SD=2.04) and all 8 patients scored <13.

#### Asthma quality of life results (Mini-AQLQ)

In this study, 5/8 participants scored 4 or less, with an overall mean score of 3.49 (SD=0.94) reported.

### Patient and practitioner feedback

A total of 9 service users and 12 members of staff were included in the PPI forums. The following themes were identified during the meetings:

#### Recognition of respiratory comorbidity

Service users demonstrated awareness that the inhalation of heroin, crack cocaine and cannabis could impact on their breathing and respiratory health. For example, one service user noted how asthma-related symptoms were heightened when inhaling heroin, leading to repeated use of reliever inhalers.

When I'm smoking heroin, it just makes it (asthma) ten times worse.

Patterns of reliever (blue) and preventer (brown) inhaler use also supported the proposal that this group were at risk of developing respiratory health problems.

A lot of them have got asthma pumps, nearly everyone, but not the brown ones.

The potential danger of inhaling the additive agents often mixed with heroin was also recognised as an issue, as was the storage of the substance, with service users highlighting the fact that the heroin they inhaled was often stored in used fuel containers.

The dealers hide the heroin in petrol or diesel containers so it always stinks of petrol/diesel when you inhale it.

The impact of poor housing conditions was also recognised by service users as a potential contributing factor to poor respiratory health.

A lot of people with addiction problems as well, they have housing problems, and they get put in all sorts of places. Rising damp, spores, yeah? Gets in your lungs.

An awareness of the burden of respiratory health was also recognised by healthcare professionals, with an overall perception that more focus is required on respiratory health and whole patient care in general.

#### Barriers to accessing to health services

The need for further research into the screening of OMUs in specialist settings was further highlighted by the barriers to accessing primary healthcare services for service users. With regards to their lifestyle, service users highlighted frequent re-locating as a key barrier to accessing care, the attitudes of healthcare professionals and the current operating systems in place. With regard to their lifestyle, service users highlighted frequent relocating as a key barrier to accessing care:

Yeah, a lot of us have that in common, move a lot, 2 doctors a year, for the last 5 years… at least.

Several service users described dismissive attitudes of healthcare practitioners, leading to reluctance to access such services.

I went to the doctor, um, about my heart, about this problem here, about breathing…and they said oh do you inject heroin, I'm like no, and I was actually like clean of all opiates, for over 10 months, and so I said that but of course, they see your history and they don't believe you.

Finally staff members reflected on the difficulties service users experience in accessing healthcare services, with suggestions that service users often found the telephone booking systems for GP services difficult to use and that the narrow time frame available to book appointments discouraged service users from accessing primary care services. It was once again proposed that such systems formed a considerable barrier to care when combined with the desire for an instant appointment and the chaotic lifestyle associated with OMUs who accessed services.

### Willingness to use screening

With regard to the potential benefit of respiratory health screening programmes, service users implied a willingness to use such services in substance misuse clinics and pharmacies and to further participate in further research in this field:

Yes people do want to think about improving their long term health and when healthcare workers offer ways to help them do this, it is appreciated…

Some concerns were raised over the capacity of such settings to offer additional care:

Queues too big in the big pharmacies, but going to a small pharmacy, I think they might have the time.

Service users also stated that the use of incentives was not a necessary requirement to encourage participation in research, as the spirometry and lung questionnaires were not invasive or too time consuming. The detrimental impact of incentivising participants was also noted by the staff. In this instance, it was suggested that examples of patients self-referring in order to receive the £10 voucher for taking part could cause a disruption to the service. The high number of potential participants, which exceeded expectations, highlighted the potential widespread acceptability of such screening services. However, it also demonstrated the potential strain placed on resources in community settings and the potential negative impact this could have on the patient's experience of care, if appropriate resource planning is not undertaken. For example, the staff reported that although they were happy to facilitate the research, limited availability of space hindered recruitment.

## Discussion

Findings from the current study suggest that it is feasible to screen for respiratory health problems in a clinic population of OMUs. These screening programmes could lead to a better understanding of the prevalence of the issue, the contributing factors and help improve access to proactive respiratory healthcare for OMUs. The acceptability of respiratory screening in the OMU population is demonstrated through the high rates of participation within a 5-week period and the wealth of data collected from participants. For example, service users were willing to complete a survey including four validated scales relating to respiratory health and quality of life and items measuring sociodemographic status and all participants were willing to undergo spirometry testing during the study. This willingness to undertake respiratory screening in routine settings mirrors previous findings from a study looking at COPD screening over three CRI centres, in which 129 heroin users were recruited over a 6-month period.^10^

Support for the feasibility of implementing a respiratory screening programme in substance misuse clinics was evident from the feedback presented at two separate PPI events including both healthcare professionals and service users. The discussion taking place at these events highlighted the added value of integrating practitioner and service user feedback into the research process. In addition to highlighting the importance of whole patient care in OMUs and the willingness and capacity available to deliver screening services, these forums also offered solutions on how to overcome barriers relating to practical limitations associated with research in these settings and to service access. These barriers to accessing care for this population, relating to structural issues and the stigma of addiction, have also been reported in the wider literature.^27^ ^28^ In this instance, it has been suggested that the use of appointment reminders and a degree of flexibility over appointments could improve access to care and treatment retention. Feedback from service users also provided further evidence of poor respiratory health in OMUs as a result of substance misuse and associated environmental factors, such as poor housing conditions. These concerns over the impact of poor housing on respiratory health are substantiated by findings suggesting factors such as damp and mould are determinants of developing asthma.^29^

Evidence of poor asthma control, in relation to the lack of awareness and use of preventer inhalers, was also noted from the PPI forums. Poor asthma control potentially puts this population at high risk of acute, severe exacerbations; chronic asthma and progression to COPD, with a recent national report suggesting that underprescription of reliever inhalers were evident in 80% of all asthma deaths in 2014. Evidence of poor asthma control in this group is also supported from the initial ACT scores reported in this group. Results from the LFQ and spirometry testing also suggested evidence of a respiratory health burden in OMUs. For example, LFQ scores suggested that 79% of the participants had symptoms associated with a risk of developing COPD and spirometry scores indicated 14% had potential obstructive lung disease. This potential burden once again adds further support to previous findings from the screening of heroin users across CRI services in Liverpool, in which overall, 61% of the sample were identified as showing symptoms of airway disease, with 28% of participants identified as displaying symptoms consistent with COPD.^10^ In contrast to this previous study, the current project assessed the feasibility of targeting settings solely designed for substance misusers. Furthermore, through using the ACT, quality of life measures and integrating PPI feedback, the current study provided a more in-depth understanding of the some of the implicating factors of poor respiratory health in this population, such as the barriers to respiratory healthcare, and how this impacts on this population's overall physical and mental well-being. The current study also assessed the feasibility of using TOPs data as a routine database outcome measure for research relating to comorbidities and opiate misuse. In this instance, some issues were reported in relation to missing data and the internal reliability of the scale. However, previous analysis suggests that the TOPs data are both valid and reliable.^26^ It is therefore suggested that certain items included in the TOP could be used in future research, with the impact of missing data potentially reduced if integrated into the initial data collected by the study team.

A further factor to take into consideration is the impact of polydrug use on the respiratory health of OMUs. For example, findings from the current study showed that the majority of service users had also experience of inhaling cocaine and cannabis, with previous studies demonstrating the inhalation of such substances to have a caustic effect on the lungs.^30^ ^31^ In addition to this, smoking prevalence is also especially high in this pilot sample, with all participants reporting to have smoked tobacco, and only one participant no longer a current smoker. These figures exceeded prevalence rates reported in a recent systematic review of smoking prevalence in addiction treatment programmes, which were 85.1% in OMUs.^32^ It is clear that such factors as tobacco smoking and polydrug use need to be controlled for in future screening studies in order to gain a better understanding of the overlapping effects in this population.

A number of limitations with the current study have to be noted. For example, due to the small sample size and lack of statistical analysis, the findings from this feasibility study in relation to the prevalence of poor respiratory health in this population have to be approached with caution. We were not able to collect data on non-participants and thus, although the provision of vouchers encouraged all service users to attend regardless of whether they had experienced respiratory symptoms or not, participants in this study may have differed from the general clinic population. Conversely, it could also be suggested that the sample could be biased by service users with poor respiratory health being more likely to select to take part in the study. The study may also have differed from the general clinic population with regard to the age. For example, although the average age of 37 years was reflective of recent national data from the National Drug Treatment Monitoring System, there was an under-representation of participants over the age of 40 years.^33^ For example, only 10% were above 40 years in the current study, compared with 43% reported in national data on this population. A further limitation is the ongoing debate over the appropriateness of such tools as the LFQ due to concerns over the number of false positives when used as a screening tool.^17^ There is also debate regarding the GOLD established parameters for diagnosis of COPD using spirometry testing results, with Cartwright also suggesting that there is evidence of misdiagnosis in older populations. For such reasons, Cartwright^17^ concluded to recommend against population screening for COPD and reported concerns over the capacity in primary and secondary care to implement such programmes. However, it has been noted that cost-effective evidence is available for the opportunistic case finding of respiratory health issues in patients with at higher risk of disease development, such as smokers and individuals aged over 35 years.^34^ In this instance, it was reported that the potential cost per life year gained is £713.16 and the potential cost per quality-adjusted life year gained is £814.56. Therefore, it is suggested that targeted programmes would be beneficial to other potential high-risk groups, such as OMUs. This current study has suggested that settings such as community substance misuse clinics hold sufficient capacity to implement such programmes.

## Conclusions

Through the integration of pilot and feasibility work and staff/patient engagement, the current study suggests that it is identified as an important topic to patients and practitioners and feasible to undertake the respiratory health screening of OMUs in a community substance misuse clinic. The relatively high prevalence of respiratory symptoms, as measured by the LFQ and abnormal spirometry make a case for a larger research screening programme for respiratory health burden in these settings, including spirometry with reversibility testing order to establish the population prevalence of respiratory health problems such as asthma and COPD in OMUs.

In line with this proposal, work is currently being undertaken by a UK Academic Health Sciences Network sponsored study team looking at whether OMUs with comorbid asthma are at a higher risk of poorly controlled asthma when compared with a population of non-OMUs with asthma. It is also proposed that future research should investigate place and mode of delivery of proactive respiratory healthcare to a high-risk group of OMUs with comorbid respiratory health problems such as asthma.

## References

[1] Strang J, Griffiths P, Gossop M Heroin smoking by ‘chasing the dragon’: origins and history. Addiction 1997;92:673–83. 10.1111/j.1360-0443.1997.tb02927.x 9246796

[2] Krantz AJ, Hershow RC, Prachand N Heroin insufflation as a trigger for patients with lifethreatening asthma. Chest 2003;123:510–17. 10.1378/chest.123.2.510 12576374

[3] Hughes S, Calverley PM Heroin inhalation and asthma. BMJ 1988;297:1511–12. 10.1136/bmj.297.6662.1511 3147049 PMC1835195

[4] Cygan J, Trunsky M, Corbridge T Inhaled heroin-induced status asthmaticus: five cases and a review of the literature. Chest 2000;117:272–5. 10.1378/chest.117.1.272 10631229

[5] Royal College of Physicians. *Why asthma still kills: the National Review of Asthma Deaths (NRAD) Confidential Enquiry report*. London: RCP, 2014.

[6] Walker PP, Thwaite E, Amin S The association between heroin inhalation and early onset emphysema. Chest 2015;148: 1156–63. 10.1378/chest.15-0236 26020453

[7] Department of Health. An outcomes strategy for chronic obstructive pulmonary disease (COPD) and Asthma in England. London: The Stationery Office, 2011:1–56.

[8] Healthcare Commission. Clearing the air: A national study of chronic obstructive pulmonary disease. London: Commission for Healthcare Audit and Inspection, 2006.

[9] Pierce M, Bird SM, Hickman M National record linkage study of mortality for a large cohort of opioid users ascertained by drug treatment or criminal justice sources in England, 2005–2009. Drug Alcohol Depend 2015;146:17–23. 10.1016/j.drugalcdep.2014.09.782 25454405 PMC4294586

[10] Lewis-burke N, Vlies B, Wooding O A screening study to determine the prevalence of airway disease in heroin smokers. COPD 2016;13:333–8. 10.3109/15412555.2015.1082999 26701201

[11] De Alba I, Samet JH, Saitz R Burden of medical illness in drug- and alcohol-dependent persons without primary care. Am J Addict 2004;13:33–45. 10.1080/10550490490265307 14766436

[12] Department of Health. A summary of the health harms of drugs. London: The Stationery Office, 2011.

[13] Beynon C, Stimson G, Lawson E Illegal drug use in the age of ageing. Br J Gen Pract 2010;60:481–2. 10.3399/bjgp10X514710 20594437 PMC2894376

[14] Rabe KF, Hurd S, Anzueto A Global strategy for the diagnosis, management, and prevention of chronic obstructive pulmonary disease GOLD executive summary. Am J Respir Crit Care Med 2007;176:532–55. 10.1164/rccm.200703-456SO 17507545

[15] National Institute for Health and Care Excellence. Asthma: Quality Standard. London: NICE, 2013.

[16] National Institute for Health and Care Excellence. Chronic obstructive pulmonary disease in over 16s: diagnosis and management. London: NICE, 2010. 31211541

[17] The UK National Screening Committee (UK NSC). An evaluation of screening for COPD against the National Screening Committee criteria. 2012.

[18] Gibbons E, Fitzpatrick R A structured review of patient-reported outcome measures for people with asthma: an update 2009. Oxford: Nuffield Department of Population Health, 2009.

[19] Davies N, Gibbons E, Fitzpatrick R A structured review of patient-reported outcome measures for COPD: an update 2009. Oxford: Nuffield Department of Population Health, 2009.

[20] Brooks R EuroQol: the current state of play. Health Policy 1996;37:53–72. 10.1016/0168-8510(96)00822-6 10158943

[21] Yawn BP, Mapel DW, Mannino DM Development of the Lung Function Questionnaire (LFQ) to identify airflow obstruction. Int J Chron Obs Pulmon Dis 2010;5:1–10. PMC2846155 20368906

[22] Nathan RA, Sorkness CA, Kosinski M Asthma, rhinitis, other respiratory diseases. Development of the Asthma Control Test: a survey for assessing asthma control. J Allergy Clin Immunol 2004;113:59–65. 14713908 10.1016/j.jaci.2003.09.008

[23] Juniper EF, Guyatt GH, Cox FM Development and validation of the Mini Asthma Quality of Life Questionnaire. Eur Respir J 1999;32:8. 10.1034/j.1399-3003.1999.14a08.x 10489826

[24] Juniper EF, Guyatt GH, Epstein RS Evaluation of impairment of health related quality of life in asthma: development of a questionnaire for use in clinical trials. Thorax 1992;47: 76–83. 1549827 10.1136/thx.47.2.76 PMC463574

[25] van der Molen T, Willemse BW, Schokker S Development, validity and responsiveness of the Clinical COPD Questionnaire. Health Qual Life Outcomes 2003;10:1–10. 10.1186/1477-7525-1-13 PMC156640 12773199

[26] Marsden J, Farrell M, Bradbury C Development of the treatment outcomes profile. Addiction 2008;103:1450–60. 10.1111/j.1360-0443.2008.02284.x 18783500

[27] Notley C, Maskrey VL, Holland R The needs of problematic drug misusers not in structured treatment—a qualitative study of perceived treatment barriers and recommendations for services. Drugs Educ Prev Policy 2012;19:40–8. 10.3109/09687637.2011.570384

[28] Notley C, Blyth A, Maskrey VL The experience of long-term opiate maintenance treatment and reported barriers to recovery: a qualitative systematic review. Eur Addict Res 2013;19:287–98. 10.1159/000346674 23652159

[29] Quansah R, Jaakkola MS, Hugg TT Residential dampness and molds and the risk of developing asthma: a systematic review and meta-analysis. PLoS ONE 2016;7:1–9. 10.1371/journal.pone.0047526 PMC3492391 23144822

[30] Nguyen ET, Silva CIS, Souza CA Pulmonary complications of illicit drug use: differential diagnosis based on CT findings. J Thorac Imaging 2007;22:199–206. 10.1097/01.rti.0000213567.86408.19 17527131

[31] Tashkin DP Airway effects of marijuana, cocaine, and other inhaled illicit agents. Curr Opin Pulm Med 2001;7:43–61. 10.1097/00063198-200103000-00001 11224724

[32] Guydish J, Passalacqua E, Pagano A An international systematic review of smoking prevalence in addiction treatment. Addiction 2016;111:220–30. 10.1111/add.13099 26392127 PMC4990064

[33] Public Health England. Adult substance misuse statistics from The National Drug Treatment Monitoring System (NDTMS). London: 2015.

[34] Centre NCG. Chronic obstructive pulmonary disease: management of chronic obstructive pulmonary disease in adults in primary and secondary care. London: 2010. 22319804

